# Layers of human brain activity: a functional model based on the default mode network and slow oscillations

**DOI:** 10.3389/fnhum.2015.00248

**Published:** 2015-04-29

**Authors:** Ravinder Jerath, Molly W. Crawford

**Affiliations:** Augusta Women's CenterAugusta, GA, USA

**Keywords:** default mode network, consciousness, limbic system, cardiorespiratory oscillations, corticothalamic processing, slow oscillations

## Introduction

The complex activity of the human brain makes it difficult to get a big picture of how the brain works and functions as the mind. We examine pertinent studies, as well as evolutionary and embryologic evidence to support our theoretical model consisting of separate but interactive layers of human neural activity. The most basic layer involves default mode network (DMN) activity and cardiorespiratory oscillations. We propose that these oscillations support other neural activity and cognitive processes. The second layer involves limbic system activity accompanied by corresponding changes in cardiorespiratory oscillations. The third layer consists of corticothalamic processing and involves higher cortical functions including awareness, cognition, and consciousness. These layers interact to form the complex neural activity of the human brain. Examining the origins and relationships of various neural and physiologic oscillations may provide better understanding of human neurophysiology and consciousness.

## Default mode network and cardiorespiratory rhythms

A number of EEG and fMRI studies in mammals indicate spontaneous low frequency oscillations in cerebral activity at <0.1 Hz represent a fundamental component of brain activity (Birn et al., 2006; Raichle and Snyder, 2007). Areas involved in this intrinsic activity include the posterior cingulate cortex/precuneous, medial prefrontal cortex, and bilateral temporo-parietal junction, are known as the “default mode network” (Buckner et al., 2008). The DMN is a resting state network that is active during passive moments and de-activated when one engages in a mental task (Brewer et al., 2011), however, the majority of energy utilized in the brain can be attributed to DMN activity (Fox et al., 2005).

Non-neural peripheral oscillations such as cardiac and respiratory waves influence resting state network oscillations (Tong et al., 2013) and the slow hemodynamic oscillations associated with resting state networks may underlie much faster neural oscillations (Yuan et al., 2012). In addition, infraslow 0.01–0.1 Hz oscillations, which have been shown to be prominent and significant during cognitive tasks, correlate highly with faster (1–40 Hz) neuronal oscillations (Monto et al., 2008), suggesting a relationship between the fast and slow oscillations, consistent with our model in which we propose DMN and cardiorespiratory oscillations may underlie fast oscillations. Another study found synchronization between 0.07 and 0.13 Hz slow prefrontal oxyhemoglobin oscillations and alpha/beta oscillations in the resting brain, suggesting that these slow oscillations can modulate excitability in cortical areas of the resting state network (Pfurtscheller et al., 2012). One study found that heart rate interval has a negative correlation with alpha power, suggesting that variations in heart rate may indicate important information about the default state and resting state networks (De Munck et al., 2008).

Respiration, heart rate, and arterial blood pressure synchronize and oscillate at 0.15 Hz during resting states, which is associated with decreased levels of reticular activity in the brainstem (Perlitz et al., 2004). Low frequency fluctuations, indicative of DMN activity, correlate with respiratory variations, however, the effect of respiratory and cardiac fluctuations in DMN analysis is poorly understood (Birn et al., 2008). In fact, these hemodynamic fluctuations are considered artifacts (Birn et al., 2008; Chang and Glover, 2009) and several methods have been developed to reduce these cardiac and respiratory signal fluctuations. Low-frequency fluctuations (<0.1 Hz) that occur throughout the body may suggest the existence of underlying network dynamics that emerge from intrinsic brain processes. For example, slow oscillations dominate physiological rhythms, suggesting that resting state networks may not be neural in origin and may merely reflect changes in respiration and/or hemodynamics. However, a study by Yuan et al. found a significant correlation between slow fluctuations of alpha EEG power, respiration, and BOLD signals during eyes-closed resting (Yuan et al., 2013), leading the researchers to suggest that alpha waves, respiratory oscillations, and BOLD signals are linked and may have a common neuronal origin (Yuan et al., 2013). Large amplitude 0.1 Hz slow sinusoidal hemodynamic cortical oscillations have been reported in human fMRI's but they are rarely considered or even noted (Rayshubskiy et al., 2014). Little research has been done on these oscillations but it is known that they are not to the result of blood pressure oscillations (Rayshubskiy et al., 2014). We propose that the interactions between the default mode network and cardiorespiratory oscillations are significant and these oscillations should not be considered as strictly noise, in fact these oscillations may underlie faster neural oscillations.

It is inherently difficult to establish a direct cause and effect relationship regarding the effects of cardiorespiratory oscillations due to the homeodynamic feedback mechanisms involved, however, the possible modulatory effects become more apparent during certain states of heightened cardiorespiratory synchronization such as during slow-wave sleep, (Jerath et al., 2014b), mindfulness meditation, and pranayama (Jerath et al., 2014a). In addition, cardiorespiratory oscillations and cardiorespiratory coupling may influence emotion states and the autonomic nervous system via modulatory feedback (Jerath and Crawford, in press). Slow wave power and cardiorespiratory coupling are tightly coupled during slow wave sleep, suggesting the integration of neural and cardiorespiratory system control during sleep (Thomas et al., 2014). A study on heart rate variability during sleep found that changes in heart rate variability occurred 1–2 min before changes in neural activity (Otzenberger et al., 1997). In addition, a study on childhood epilepsy found that heart rate changes preceded 70% of partial and focal seizures (Jansen et al., 2013). Though these studies cannot prove causative effects, we believe they establish a need for further research needs to be done to elucidate the role of cardiorespiratory oscillations in neural activity.

Although little research has been done on the possible relationship between respiratory and DMN oscillations, extensive research has been done on the DMN's involvement in consciousness. For example, the resulting unconsciousness that occurs during various types of epileptic seizures, may be due to inhibition of arousal systems that normally maintain the default mode network during awake states (Danielson et al., 2011). It has also been found that connectivity between the thalamus and anterior cingulate decreases during vegetative states and increases when consciousness is recovered (Laureys et al., 2000) while arousal structures like the reticular formation, hypothalamus, and basal forebrain sustain relatively normal levels of activity during vegetative states (Laureys et al., 2004). Levels of DMN connectivity also reflect the level of consciousness in brain-damaged (Vanhaudenhuyse et al., 2010), vegetative, and minimally conscious patients and correlate with behavioral signs of awareness (Fernandez-Espejo et al., 2012). These studies suggest that the DMN may be an essential neural network involved in consciousness and may support higher processing.

## Embryologic development of DMN, resting state networks, and associated cardiorespiratory interactions

Resting-state networks (RSN) have been detected in infants between 20 and 36 gestational weeks (Schopf et al., 2012). Another study in preterm infants found recognizable but fragmented RSN activity at 30 weeks and complete adult patterns at full-term (Doria et al., 2010). In addition, when complete resting state networks were present at full-term, several were integrated with thalamic activity while auditory and visual networks appeared completely developed (Doria et al., 2010). These studies suggest that RSNs develop rapidly during the third trimester and are likely formed before higher cognitive abilities develop (Doria et al., 2010). The appearance of RSNs when corticothalamic networks are developing suggests that RSNs may be linked to corticothalamic activity (Doria et al., 2010), however, some studies have found that the DMN does not completely develop in utero (Doria et al., 2010; Smyser et al., 2010).

## Limbic system, cognitive processing, and cardiorespiratory rhythms

The mesencephalon, diencephalon, and telencephalon form early on in embryological development and regions of these structures form the limbic system (Nieuwenhuys et al., 1996). The limbic system has been proposed to have evolved before higher cortical areas, with the brainstem forming first, followed by the limbic system, and then the cortex, along with some subcortical structures (Holden, 1979). These areas are known colloquially as the reptile brain, old mammalian brain, and new mammalian brain, respectively. According to current research, this model of brain structure evolution appears probable; however, the colloquial terms are misleading. Common ancestors of reptiles and mammals had well-developed limbic systems (Bruce and Neary, 1995) so the use of terms like “reptilian” and “old mammalian brain” are inaccurate. This evolutionary model is consistent with our hypothesis in which the DMN, limbic system, and cortical areas make up separate but interactive layers of neural activity.

The limbic system, like the DMN, is also closely tied to cardiorespiratory activity and oscillations (Masaoka and Homma, 2005; Homma and Masaoka, 2008). The modulating effect that emotions and the limbic system have on respiration and vice versa has been well documented (Masaoka and Homma, 2005; Homma and Masaoka, 2008). There are many different oscillations that are indicative of specific processes in the brain and are also associated with the DMN. For example, alpha and theta oscillations which have been associated with cognition and memory (Klimesch, 1999), interact with the DMN. Alpha oscillations have been shown to overlap with the DMN (Knyazev et al., 2011) and the DMN has been highly correlated with the possible synchronization of internal mental processes by alpha oscillations (Knyazev et al., 2011). Gamma oscillations have been associated with a variety of cognitive tasks involving learning (Popescu et al., 2009) and memory (Howard et al., 2003) and suppression of gamma power within the DMN is correlated with task complexity and performance (Ossandon et al., 2011). Beta oscillations have shown a strong correlation with DMN connectivity (Hlinka et al., 2010; Neuner et al., 2014). These studies illustrate the DMN's role in modulating and interacting with fast oscillations.

## Layers of neural activity

**Figure 1:**
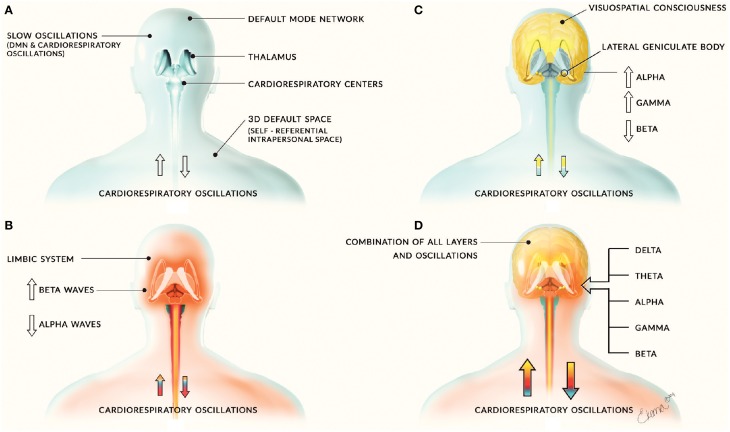
**Layers of neural activity**. Panel **(A)** depicts the first layer of neural activity that includes slow oscillations from the default mode network and cardiorespiratory oscillations. This forms the basis for all other layers of neural activity and is depicted by the blue coloring. Panel **(B)** includes limbic activity and is depicted by the red coloring. It is associated with increased beta waves and decreased alpha waves. Panel **(C)** includes corticothalamic feedback loops involved in cognitive and consciousness processes and is depicted by yellow coloring. This layer is associated with increased alpha and gamma waves and decreased beta waves. These layers combine to form panel **(D)**, the sum of human neural activity consisting of all neural and physiological oscillations. The combination of this activity is denoted by the combination of the blue, red, and yellow layers.

We propose the system of complex and dynamic neural activity and oscillations can be divided into separate layers of distinct activity. The first layer consists of interactions between the DMN and cardiorespiratory oscillations, which allow the mind and body to behave as a unified space. These slow oscillations may allow for the formation of fast neural oscillations and subsequent layers of neural activity (Figure 1A). We also propose that the DMN may play a fundamental role in neural network connectivity. In addition, studies on the involvement of the DMN in consciousness suggest that the DMN plays an integral role in consciousness processes. We propose that the DMN may be involved in the integration of external and internal sensory input into consciousness experience, as well as creating an oscillatory neural framework in which this processed sensory information rises to consciousness arises. For example, the anterior insular cortex has been shown to be involved in integration of multisensory processing (Chen et al., 2015) and a recent study found that the posterior cingulated cortex was a cortical functional hub for a large scale DMN network involved in functional connectivity and integration of local systems (De Pasquale et al., 2013). We propose that the processing and integration of sensory information may be supported by the default mode network, resting state networks, and associated cardiorespiratory oscillations. However, the specifics of this consciousness model are beyond the scope of this paper; therefore; for an in depth discussion of the full consciousness model (see Jerath and Crawford, 2014).

Ninety percent of the energy used by the brain is devoted to intrinsic activity rather than activity evoked by stimuli (Raichle and Snyder, 2007) and the majority of energy utilized in the brain can be attributed to DMN activity (Fox et al., 2005), suggesting that DMN baseline activity may support and maintain other activity within the brain. The sheer amount of neural energy utilized by the DMN, the activation of the DMN at rest, and studies on the involvement of the DMN in consciousness, make the DMN a likely candidate for maintaining a neural framework on which other oscillatory activity builds and creating an active neural space which is filled in by higher processing to give rise to conscious experience. We propose that along with the DMN's role in integration and providing a framework for higher processing, energy utilized by the DMN may be used to create and maintain the framework on which consciousness is built. There are inherent difficulties in deciphering cardiorespiratory and neural oscillations and establishing direct evidence of a cause and effect relationship therefore extensive research is needed to support these hypotheses and elucidate the role of the DMN and cardiorespiratory oscillations, as well as their role in consciousness and neural activity as a whole.

If this model of layers of neural activity is proven correct it could have numerous applications for improving the study of the brain and deciphering neural activity. Research on the possible link between neural activity, cardiorespiratory oscillations, and the role of the DMN could reveal insights about higher processing such as those involved in awareness, memory, cognitive performance, and consciousness and may also give us insights into how to interpret EEG activity.

## Conclusion

We propose that neural activity can be categorized into separate layers consisting of (1) the default mode network and cardiorespiratory oscillations, (2) limbic activity (Figure 1B), and (3) cortical activity involved in consciousness and cognitive processes (Figures 1C,D). The DMN and cardiorespiratory oscillations may support other neural activity and create a framework on which other neural activity and consciousness arises. The close relationship of major peripheral oscillations such as cardiac and respiratory rhythms with fast and slow oscillations of the central nervous system helps to illustrate global dynamic processes and interactions that occur within the human body, which may influence neural activity. Further research on this model would greatly further our understanding of the DMN and how the brain works as a whole, including insights into the way we interpret and examine EEG activity. Our model draws evidence from relevant studies, along with evolutionary and embryologic evidence to propose that DMN, salient networks, respiratory, and hemodynamic oscillations play a fundamental role in supporting neural activity and are involved in energy dynamics in the brain. However, little research has been done to study this relationship and the role of the DMN is largely unknown, therefore extensive research is needed to explore this model.

### Conflict of interest statement

The authors declare that the research was conducted in the absence of any commercial or financial relationships that could be construed as a potential conflict of interest.
